# Cultivable Actinobacteria First Found in Baikal Endemic Algae Is a New Source of Natural Products with Antibiotic Activity

**DOI:** 10.1155/2020/5359816

**Published:** 2020-07-27

**Authors:** Denis V. Axenov-Gribanov, Daria V. Kostka, Ulyana А. Vasilieva, Zhanna M. Shatilina, Maria E. Krasnova, Ekaterina V. Pereliaeva, Elena D. Zolotovskaya, Maria M. Morgunova, Olga O. Rusanovskaya, Maxim A. Timofeyev

**Affiliations:** ^1^Irkutsk State University, Karl Marx St. 1, 664003 Irkutsk, Russia; ^2^Irkutsk Regional Clinical Advisory and Diagnostic Center, Baykalskaya Str., 109 Irkutsk, Russia; ^3^Siberian Institute of Plant Physiology and Biochemistry, 132 Lermontov Str., 664033 Irkutsk, Russia; ^4^Baikal Research Centre, Lenin Str. 21, 664003 Irkutsk, Russia

## Abstract

Inadequate use of antibiotics has led to spread of microorganisms resistant to effective antimicrobial compounds for humans and animals. This study was aimed to isolate cultivable strains of actinobacteria associated with Baikal endemic alga *Draparnaldioides baicalensis* and estimate their antibiotic properties. During this study, we isolated both widespread and dominant strains related to the genus *Streptomyces* and representatives of the genera *Saccharopolyspora, Nonomuraea, Rhodococcus*, and *Micromonospora*. For the first time, actinobacteria belonging to the genera *Nonomuraea* and *Saccharopolyspora* were isolated from Baikal ecosystem. Also, it was the first time when actinobacteria of the genus *Nonomuraea* were isolated from freshwater algae. Some rare strains demonstrated activity inhibiting growth of bacteria and yeasts. Also, it has been shown that the strains associated with Baikal alga *D. baicalensis* are active against both Gram-positive and Gram-negative bacteria. According to this study and previously published materials, diversity of cultivable actinobacteria and rare strains isolated from *D. baicalensis* is comparable to that of cultivable actinobacteria previously isolated from plant sources of Lake Baikal. Also, it exceeds the cultivable actinobacteria diversity previously described for macroinvertebrates, water, or sediments of Lake Baikal. The large number of rare and active strains associated with the endemic alga *D. baicalensis* could be the promising sources for biopharmaceutical and biotechnological developments and discovery of new natural compounds.

## 1. Introduction

Screening, synthesis, and production of biologically active compounds and natural products represent one of the most important research trends in biopharmaceutical studies all over the world [1]. The necessity to develop new drugs is induced by rapidly growing multidrug resistance of microorganisms to antibiotics introduced into medical and clinical practice [2, 3]. However, the search and development of new biologically active compounds is complicated because most ecosystems and microorganisms producing natural products are already well-analysed. This leads to the fact that the probability to find new biologically active compounds and producers in this studied communities is relatively low [4]. At the same time, the chemical modification methods and those used in bioinformatics to predict and obtain new bioactive natural products have a natural limitations. Accordingly, of particular interest is the search and development of new biologically active compounds from specific symbiotic microorganisms that inhabit ecosystems with specific evolutionary and ecological characteristics. One of the most promising places for biopharmaceutical search is unique and little studied ecosystem of the ancient Lake Baikal.

Lake Baikal is a UNESCO World Heritage site. It seems to be a unique natural laboratory characterized by particular conditions of the lake, such as low temperatures, low content of mineral and organic elements, high oxygen content in the entire water column, and the highest level of biodiversity of flora and fauna [5]. Lake Baikal is the largest center of speciation, endemism [6], and the “pantry” of the genetic resources of the world.

Inhabitants of Lake Baikal play an important role in the ecosystem stability. At the same time, inhabitants of lake presented by diverse flora and fauna are ecological niches for microorganisms. Thousands of algae species endemic to Lake Baikal are the hosts for microorganisms that can be used in human activity. Futhermore, the algae diversity is of great significance for the ecosystem.

As a primary trophic chain, algae play a major role in life of water reservoirs. Structural and functional features of this chain largely determine the appearance of a lake ecosystem [7]. Also, algocenoses are very sensitive biological indicators, and they can detect minor changes that cannot be detected using other methods [8]. In case of Lake Baikal, algae are actively involved in self-purification processes. They are a test-object for estimation of trophicity and biological productivity. Studies of systematic composition of Baikal algae began in the XIX century [9–19]. Currently, the algae flora of the lake is represented by 569 species and 162 subspecies with 35% rate of endemicity [20]. About half of these species were found in the open Baikal. Phytoplankton amounts to more than 100 true planktonic algae species. Several pictures of algae inhabiting Lake Baikal are shown in Figure S1.

However, until the present time, Baikal algae with their microbial communities were not in the focus of diversity and biomonitoring assays and did not rank as the source of new or symbiotic microorganisms species used for human needs [21, 22]. Symbiotic strains of actinobacteria are of specific interest for biotechnology because these microorganisms are rich and productive sources of genetic material for new natural products and biologically active compounds [23].

Actinobacteria belong to the group of Gram-positive bacteria with high content of guanine and cytosine in DNA. Most actinobacteria are aerobes, whereas facultative anaerobes are found only among actinobacteria with short mycelial stage. Actinobacteria are well distributed phylum of bacteria that inhabit various ecological niches, including extreme ones [24, 25].

These bacteria form associations with plants in aquatic and terrestrial environment. They participate in recycling of substances, and their distinct feature is a wide range of constructive and energy metabolism [26]. Actinobacteria play an important role in biological cycle, especially in soils [27]. Thus, bacteria related to the genus *Frankia* are well known for their plant-symbiotic associations and nitrogen-fixing abilities. Annual nitrogen accumulation in soil with participation of the associations can be around 150–300 kg/ha. Nodules are formed resulting from symbiosis between plant roots and actinobacteria related to the genus *Frankia* sp. Leghemoglobin is synthesized in these nodules. This protein protects nitrogenase against excessive amounts of molecular oxygen. Also, the role of the Microbacteriaceae family in *Arabidopsis thaliana* roots [28] was hypothesized.

In Baikal region, there were only single studies focused on assessment of cultivated actinobacteria diversity in high-plant communities. Thus, in one of the recent studies conducted by Axenov-Gribanov et al. [29], a variety of actinobacteria in *Pinus sylvestris* pollen was estimated. The authors showed that the taiga forest of Baikal Siberia has not been well studied, and its actinobacteria population remains undescribed. At the same time, diversity of cultivated communities of actinobacteria symbiotic to Baikal dominant endemic algae was not assessed, while ecology and lifestyle of this group of organisms are of interest for both fundamental and applied studies. In focus of first study for Baikal algomicrobial communities, here we aimed to isolate cultivable strains of actinobacteria associated with endemic alga *Draparnaldioides baicalensis* and estimate their antibiotic properties.

## 2. Materials and Methods

### 2.1. Sampling Sites and Isolation of Actinobacteria

During the study, pure cultures of actinobacteria were isolated from dominant freshwater endemic alga *Draparnaldioides baicalensis*. *D. baikalensis* is a freshwater filamentous green alga from the genus *Draparnaldioides*, the member of family Chaetophoraceae, order Chaetophorales, and class Chlorophyceae. The alga *D. baicalensis* (Figure S2) has a short period of vegetation. This species dominates in the summer-autumn period in the littoral zone at depths of 2.5–3.0 to 6–10 m. The thallus species is bush type. Height of the thallus is 15–35 cm; it attaches to stones. *D. baicalensis* is characterized by a powerful mucous case to protect the alga from the wave action. It has a reticulate-perforated chloroplast occupying the entire height of the cell [30, 31]. Due to ability of this alga, in the nearest future, it will be a finished complex study of chemical analysis in focus of secondary metabolism and algomicrobial interactions.

The alga samples were collected near the village Bolshie Koty (Southern Baikal, 51.902940, 105.071179) in 2018. Algal strands were washed in sterile Baikal water, then rinsed with 70% ethyl alcohol 3 times for 15 seconds, and rinsed with sterile water again 3–5 times for 30 seconds, alternatively [32, 33]. The biological material (*n* = 10, average weight is 10 g) was collected using sterile tweezers and placed into sterile Falcon-tubes, homogenized with glass pestle directly in Falcon-tubes, and stored in 3 volumes (average volume, 30 ml) of sterile 20% glycerol in a freezer at −20°C during 1-2 weeks before plating on solid nutrient media.

Actinobacteria strains were isolated by triplicate plating of 100 *μ*L of each sample on solid nutrient media. Because the current study was a primary for this source, we chose standard and typical conditions for isolation and cultivation of actinobacteria [34]. To isolate the actinobacteria strains, we used mannitol soy flour agar (MS) [34] and actinomycete isolation agar (Difco) supplemented with antibiotics cycloheximide (50 *μ*g/mL) and phosphomycin (100 *μ*g/mL). Also, aliquots of collected samples (500 *μ*L) were preheated for 5 min at 50°C to activate spore germination and inactivate vegetative cells of other bacteria. The plates were incubated for 30 days at 28°C and assessed for emergence of actinobacteria colony every day. Actinobacteria-like strains were selected based on the colony morphology: solid density of colonies, growth inside the agar media, and steady border of the colonies [34]. The colonies were transferred from the primary plates to fresh MS plates. Pure cultures were obtained for all the colonies identified as actinobacteria on the primary plates. Several isolated strains were deposited in the Russian Collection of Agricultural Microorganisms (RCAM), St. Petersburg, Russia.

### 2.2. 16S rRNA Gene Sequencing and Phylogenetic Analysis

For isolation of total DNA, the strains were cultivated in 10 mL of TSB medium in shake flasks at 28°C for 3 days at 180 rpm. The total DNA was isolated with the salting out procedures as described in [34]. To identify the isolates, the 16S rRNA gene was amplified by PCR with the actinobacteria-specific primers: F-Act-235(CGC GGC CTA TCA GCT TGT TG) and R-Act-878(CCG TAC TCC CCA GGC GGG G) [35]. The PCR reaction was performed using the ScreenMix 5X PCR kit and included all the necessary components of PCR (high-performance Taq DNA polymerase, a mixture of deoxynucleoside triphosphates, Mg^2+^, PCR buffer, and dyes (Kat. PK041L, Evrogen, Russia. http://www.evrogen.com). PCR was performed in a T-Gradient thermocycler (Biometra, Germany) in the volume of 25 *μ*L. The PCR parameters were as follows: initial denaturation at 95°C for 5 min, followed by 25 cycles of 95°C for 40 sec, 49–52°C for 25 sec, and 72°C for 110 sec, and final elongation at 72°C for 5 min [36].

The PCR products were purified using Cleanup Standard Column Extraction kit (Kat. BC022, Evrogen, Russia) and sequenced using actinobacteria-specific primers. The PCR product mixture with amplification primers was sent to the Syntol company (Moscow, Russia) for sequencing the PCR products with the Sanger method [37]. Forward and reverse sequences were assembled with Bioedit software (version 7.2.5). The obtained sequences were deposited in the GenBank with the following numbers: MH393589–MH393597 (Table 1).

For phylogenetic analysis, the sequences were aligned using MEGA software (version 7.0) [38]. The evolutionary history was inferred using the maximum parsimony method. Percentage of replicate trees, where the associated taxa clustered together in the bootstrap test (1000 replicates), is shown next to branches [39]. Bacterium of the species *Bacillus licheniformis*, often found in microbial communities of the various plants rhizosphere, was used as outgroup.

### 2.3. Cultivation and Extraction

The isolated strains were cultivated in 30 mL of production medium in 250 mL shake flasks with baffles for 7 days at 28°C at 180 rpm shaking rate [34]. Three different liquid media were chosen to estimate primary antibiotic activity. These media are NL19 (soy flour 20 g, D-mannitol–20 g, tap water 1 L, and pH 7.2), SG (glucose 20 g, glycerol 10 g, soytone 10 g, CaCO_3_ 2 g, CoCl_2_ 0.001 g, pH 7.2, distilled water 1 L, and pH 7.2), and SM17 medium (glucose 2 g, glycerol 31.7 ml, soluble starch 2 g, soy flower 5 g, peptone 5 g, yeast extract 5 g, NaCl 5 g, CaCO_3_ 2 g, pH 6.4, tap water 1 L, pH 7.2).

The grown cultures were centrifuged at 3.000 g for 10 min to separate the biomass and cultural liquid. Then, secondary metabolites were extracted from the liquid culture with equal volume of ethyl acetate. To extract natural products from the biomass, we used 10 mL of acetone-methanol mixture (ratio 1 : 1). Extraction was performed during 1 h on a rotator at 100 rpm at room temperature. The obtained crude extracts were evaporated in vacuo using the IKA RV-8 rotatory evaporator (IKA, Germany) at 40°C and dissolved at concentration 25 *μ*g/L of methanol-DMSO mixture (ratio 1 : 1) [40].

### 2.4. Assay of Antimicrobial Activity of Isolated Strain Extracts

Antimicrobial effects of the extracted metabolites were tested using the spectrophotometry test in a microplate reader Infinite M200 (Tecan, Austria) at 610 nm wavelength. For the analysis, 96 well plates were used. 1 *μ*L of extract and 100 *μ*L of the test culture were applied to the spectrophotometer wells. Using the technique of serial dilutions, final concentration of extracts in the wells was ranged from 25 ug/L to 6.25 ug/L. The analysis was carried out in two biological and four analytical replicates at 28°C (for yeasts) and 37°C (for bacteria). Antibiotics ceftriaxone, streptomycin, and nystatin were used as positive and negative controls. Data were normalized by (1) optical density of the sterile medium and (2) optical density of the growth of test cultures in standard conditions. In these wells, the solvent used for preparations of crude extracts (mixture of methanol and DMSO) was added in the same volume. Thus, optical density of the transparent medium was equated to absence of growth of test cultures and −100% (minus means inhibition) of inhibition rate after 12–18 hours [41, 42].

Several bacterial and fungal test cultures, such as *Bacillus subtilis* ATCC 66337, *Staphylococcus carnosus* ATCC 51365, *Escherichia coli* ATCC25922, and *Saccharomyces cerevisiae* BY4742 were chosen to test antibiological properties. Test cultures were obtained from the Leibniz-Institute DSMZ-German Collection of Microorganisms and Cell Cultures (Braunschweig, Germany).

## 3. Results

### 3.1. Isolation and Phylogenetic Analysis of Actinobacteria Associated with Baikal Algae

A total of 9 strains of cultivable actinobacteria were isolated here using the selected approaches and media. BLAST analysis of nucleotide sequences aligned for the 16S rRNA gene revealed high similarity of strains between actinobacteria associated with *D. baikalensis* alga and sequences of actinobacteria strains that were previously deposited in the Genbank database (NCBI). All of the isolated strains were identified as bacteria related to phylum Actinobacteria (Table 1). It was revealed that 5 strains belonged to the genus *Streptomyces* sp. Also, one representative per each of the following genera was identified as *Sacharopolyspora* sp., *Micromonospora* sp., *Rhodococcus* sp., and *Nonomurea* sp.

Based on comparison of the nucleotide sequences of 16S rRNA gene of isolated strains aligned with the sequences of actinobacteria deposited in the Genbank (NCBI), we show that the isolated strains form tight separate clades with representatives of similar species. Separate branches of each genus are presented in Figures S3–S6. It is shown that the isolated strains related to the genus *Streptomyces* sp., *Micromonospora* sp., and *Rhodococcus* sp. do not form strict, independent, or distant clades with strains or genetic material previously found in soil or water substances, respectively.

**Figure 1:**
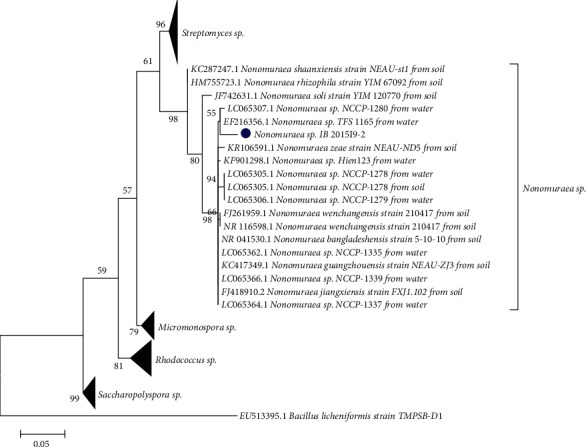
Phylogenetic tree of the genus *Nonomuraea* sp. constructed using the maximum likelihood method based on the comparison of the nucleotide sequences of 16S rRNA gene deposited in GenBank. *Note.*– Bootstrap—1000. The analysis involves 21 sequences, and the length of nucleotides is 411. The tree includes 10 strains of bacteria isolated from soil (marked “from soil”); 8 strains of bacteria isolated from water of different reservoirs (marked “from water”); and 1 strain isolated in the course of this study. The analysis was carried out in MEGA7 program. Outgroup is presented by *B. licheniformis*.

Analysis of *Nonomuraea* sp. strains showed that the strain isolated in this study forms a clade with other representatives isolated from water sources (Figure 1). Other clades are quite mixed and contain both soil and water representatives. It should be noted that, as of June 2020, no nucleotide sequences belonging to the strains isolated from algae of Lake Baikal ecosystem were deposited into the NCBI system. Thus, our study is the first to mention this genus representative in the ecosystem of Lake Baikal and its microbial-phytobenthic communities. A similar case was also noted for the isolated strain of the genus *Saccharopolyspora* sp. (Figure S6). However, in contrary to the above *Nonomuraea* sp. representatives, the NCBI system contains one nucleotide sequence for actinobacteria of this genus isolated from alga *Nostoc* sp. Like in case of *Nonomuraea* sp., the present paper is the first to mention the actinobacteria related to the genus *Saccharopolyspora* sp. in Lake Baikal ecosystem.

### 3.2. Analysis of Biological Activity of Isolated Strains

**Figure 2:**
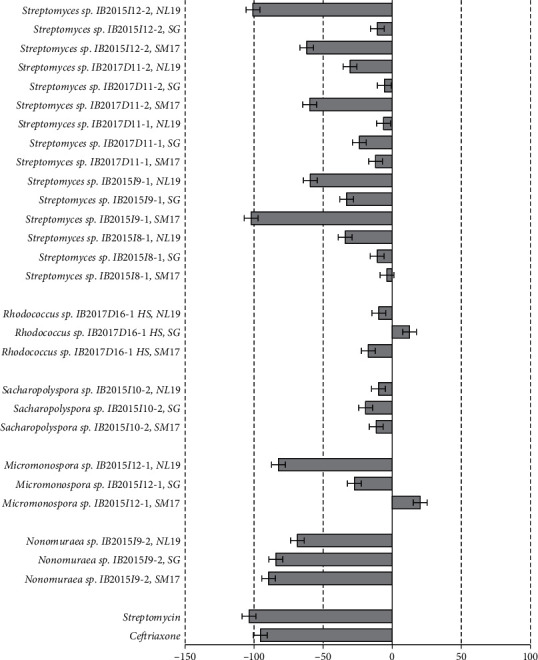
Antibacterial activity of crude extracts obtained from the cell-free liquid culture of the isolated strains at concentration 25 *μ*g/L against *St. carnosus. Note.* “-”: inhibition rate, in %, mean ± SD.

**Figure 3:**
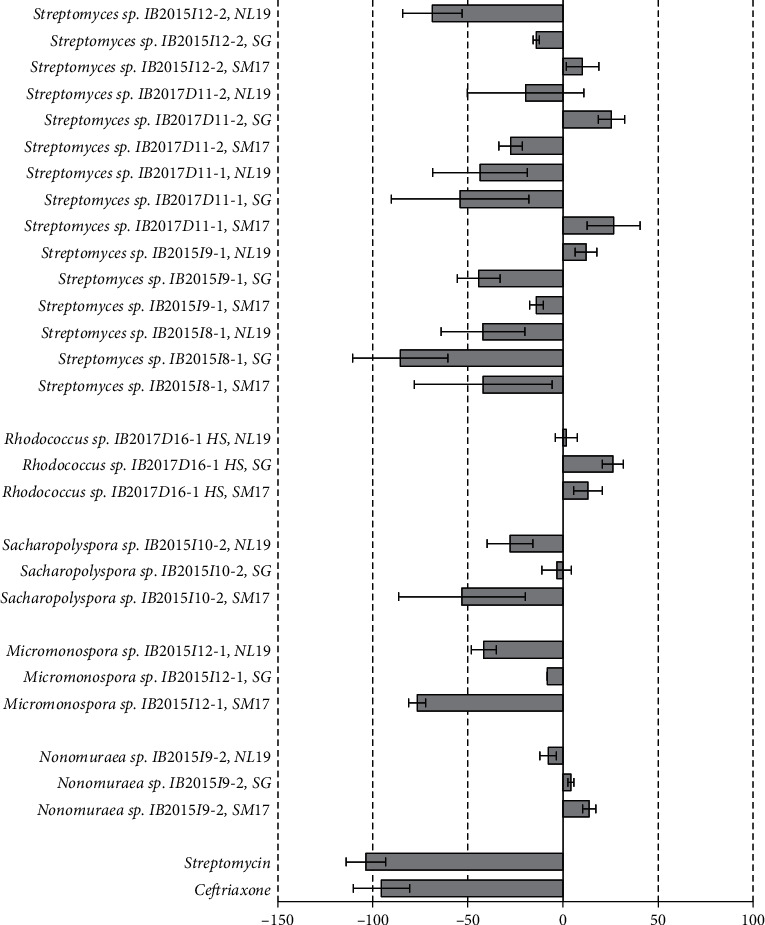
Antibacterial activity of crude extracts obtained from the cellular biomass of the isolated strains at a concentration of 25 *μ*g/L against *St. carnosus. Note.* “-”: inhibition rate, in %, mean ± SD.

**Figure 4:**
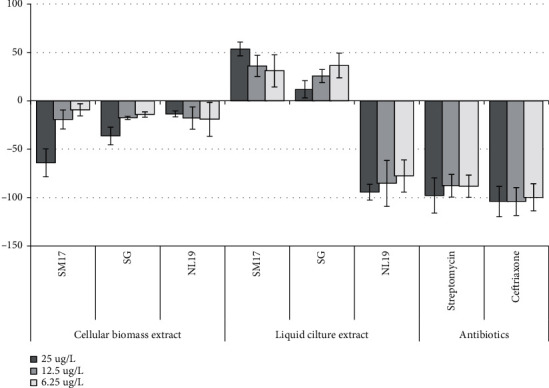
Antibacterial activity of crude extracts of strain *Micromonospora* sp. IB 2015I12-1 obtained after cultivation in different nutrient media against *B. subtilis*. *Note.* “-”: inhibition rate, in %, mean ± SD.

The materials describing the activity of the extracts of actinobacteria strains cultivated in different nutrient media against Gram-positive bacteria are presented in Figures 2–4. Antimicrobial activity of crude extracts against growth of *Staphylococcus carnosus* at 25 ug/L concentration was observed in case of cell-free liquid culture extracts (Figure 2) and cellular biomass (Figure 3) of strains *Micromonospora* sp. IB 2015I12-1, *Streptomyces* sp. IB 2015I12-2, *Streptomyces* sp. IB 2015I9-1, and rare strain *Nonomuraea* sp. IB 2015I9-2. Also, extract obtained from the cellular biomass of the strain *Streptomyces* sp. IB 2015I8-1 cultivated in SG media was characterized by high inhibition properties.

Extract of cellular biomass of the strain *Streptomyces* sp. IB 2015I9-1 did not show pronounced antagonistic activity against *St. carnosus*in at all tested concentrations (6.25–25 ug/L). However, we observed activity of crude extract of this strain when it was cultivated in SM17 medium. The observed activity can be compared with that of streptomycin and ceftriaxone antibiotics in the same concentrations (Figure S7).

Crude extract of cell-free liquid culture of the strain *Nonomuraea* sp. IB 2015I9-2 was active against *St. carnosus* culture when cultivated on all media at all concentrations. However, the crude extract of cellular biomass did not show antibiotic activity under the tested conditions (Figure S8). In case of the strain *Micromonospora* sp. IB 2015I12-1, the highest antibiotic activity was observed in extract of the cell-free liquid culture obtained by the strain cultivation in NL19 medium. Under these conditions, growth of 82 ± 8.9% cells of *St. carnosus* was inhibited. The decrease in concentration to 12.5 ug/L and 6.25 ug/L led to suppression of *St. carnosus* growth to 53–59% (Figure 4).

*Bacillus subtilis* was another culture tested in the current study. The most expressed antimicrobial activity against growth of *B. subtilis* at concentration 25 ug/L was revealed for crude extracts of the cell-free liquid culture of strains *Micromonospora* sp. IB 2015I12-1, *Streptomyces* sp. IB 2015I9-1, and *Streptomyces* sp. IB 2017D11-2. At the same time, the activity of the extracts obtained from the cellular biomass of the same strains was less (Figures S9 and S10). Among the extracts obtained from cellular biomass, the highest activity was detected for the strains *Sacharopolyspora* sp. IB 2015I10-2, *Streptomyces* sp. IB 2015I8-1, and *Streptomyces* sp. IB 2017D11-1.

Figure 4 presents the materials evaluating activity of the strain *Micromonospora* sp. IB 2015I12-1. It was shown that crude extracts of cellular biomass had weak antibiotic activity, whereas the optimal medium for extracellular synthesis of antibiotics was NL19 medium. At the same time, extracts obtained from the cell-free liquid culture of this strain grown in SM17 and SG media contributed to growth stimulation of the model test culture.

The materials describing the activity of the isolated actinobacteria extracts against Gram-negative bacteria are presented in Figures S11–S14 (supplementary). Crude extracts of the cell-free liquid culture (Figure S11) and cellular biomass (Figure S12) prepared at a concentration of 25 ug/L, obtained from several isolated strains (*Streptomyces* sp. IB 2015I9-1, *Micromonospora* sp. IB 2015I12-1, *Streptomyces* sp. IB 2015I12-2, and *Streptomyces* sp. IB 2015D11-2), inhibited the growth of *Escherichia coli*.

The materials describing the activity of the extracts of isolated actinobacteria against yeasts *S. cerevisiae* are shown in Figures S15–S18 (Supplementary). Only two isolated strains (*Streptomyces* sp. IB 2015I9-1 and *Nonomuraea* sp. IB 2015I9-2) were characterized by the pronounced activity against growth of saccharomyces (Figures S15 and S16). In case of the strain *Streptomyces* sp. IB 2015I9-1, extracts obtained from the cell-free liquid cultures of this strain during cultivation in SM17 medium were active against *S. cerevisae* growth. The activity of this strain-related extract was comparable with nystatin activity. Extracts of the cellular biomass did not show visible inhibitory activity (Figure S17 and S18). Another strain *Nonomuraea* sp. IB2015I9-2 was characterized by a weak antibiotic activity. The inhibition rate of the cell-free liquid culture extracts was less than 50%. The cellular biomass extract obtained by strain cultivation in SM17 nutrient medium showed an inhibition rate of 58 ± 7.3% against tested yeast. Crude extracts obtained by strain cultivation in another tested nutrient media caused growth stimulation of *S. cerevisae*.

## 4. Discussion

**Figure 5:**
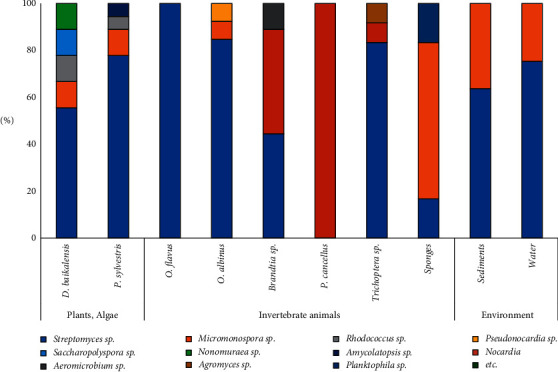
Distribution of cultivable actinobacteria isolated from different ecological groups of organisms or environment of Lake Baikal ecosystem.

During this study, we first time isolated several strains of cultivable actinobacteria from Baikal endemic alga *D. baicalensis*. We estimated their similarity with other actinobacteria and performed initial description of crude extracts antibiotic activity of bacteria cultivated in different nutrient media. According to the materials obtained in this study, we could preliminarily conclude that diversity of cultivable actinobacteria and rare genera isolated from *D. baicalensis* is comparable to that of cultivable actinobacteria that we previously isolated from the pollen of a pine species growing on the shore of the lake [29]. At the same time, according to published data, the diversity of rare genera of cultivable actinobacteria isolated here from the alga exceeds the cultivable actinobacteria diversity described for Lake Baikal macroinvertebrates [32, 43], water [44, 45], or sediments [46, 47] (Figure 5). The increased diversity of rare genera of actinobacteria in plant sources could be preliminarily explained by the chemical composition and availability of nutrients (vitamins or fatty acids) produced by plants or algae. During the analysis of nutrient media used for isolation of rare strains, we observed that specific media often include vitamins, plant extracts, and soil or humic substances [48]. Thus, the plant material seems to be “natural trap” for accumulation and attraction of rare actinobacteria species. Also, due to high organized enzymatic system of cellulose destruction in actinobacteria, this diversity could be explained by the possibility to hydrolysis of cellulose-containing cell wall of plants and algae and consumption of this source of carbon [49].

Similar to other studies conducted in the Baikal and non-Baikal regions [50, 51], the genus *Streptomyces* was found as the most common and dominant one [52, 53]. According to the phylogenetic analysis performed, representatives of Baikal actinobacteria do not form separate clades and they are highly similar to other representatives of actinobacteria related to this genus. This study identified representatives of the genera *Nonomuraea* sp. and *Saccharopolyspora* sp. in the ecosystem of Lake Baikal for the first time.

Also, a strain related to the genus *Nonomuraea* sp. was first isolated from freshwater algae. In case of the isolated strain *Saccharopolyspora* sp., only one nucleotide sequence is registered in the NCBI system as an algal-associated microbial community of *Nostoc* sp. Thus, our study is the first to mention about actinobacteria of the genus *Nonomuraea* sp. and *Saccharopolyspora* sp. living in the ecosystem of Lake Baikal.

The assessment of the role of some microorganisms and actinobacteria in plant communities is well known and has already been mentioned in the introduction chapter and in [54–57]. A lot of studies postulate the estimation of microbial diversity, associated with algae and other water-related organisms [58–60]. However, only a few studies are involved with assessment of relationship between algae and actinobacteria. As described in Zenova et al. [61], algobacterial communities represent a highly productive system. In this system, blue-green algae are the main producers of organic material. The latter can be used as a source of carbon and energy in heterotrophic microorganisms related to the community. Entering into the associations with bacteria, the blue-green algae are involved in food chain formation. Associations of actinobacteria with green algae are called actinolichen, where actinobacteria participate in the system stabilization and enhance the photosynthetic activity of algae [61]. Also, other studies have shown the stimulation of *Oscillatoria terebriformis* growth in the associated culture with the streptomyces strain. In addition, increased nitrogen fixation by *Anabaena variabilis* and higher photosynthetic activity of *O. terebriformis* in streptomyces-associated culture was recorded [62]. Michel et al. [63] showed that some algae and actinobacteria are closely related in their evolution. For example, terminal stages of cellulose and hemicellulose biosynthesis of the alga *Ectocarpus siliculosus* were inherited from actinobacteria to red alga by horizontal gene transfer [63].

**Figure 6:**
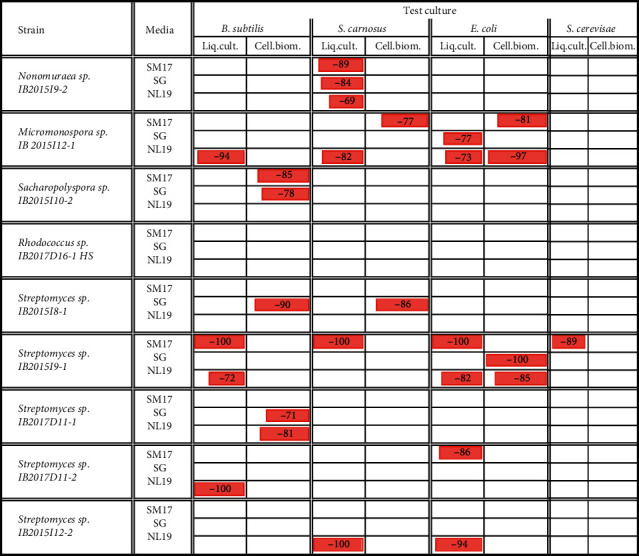
The activity of the strains isolated from endemic alga *D. baicalensis. Note.* The table shows the inhibition activity of strains in excess of 70%.

In addition to assessment of cultivated actinobacteria diversity, in this study, we demonstrated that the strains isolated from endemic algae exhibit pronounced antibiotic activity and high content of active strains, including rare strains. From the data obtained, it was established that 8 out of 9 isolated strains showed high activity with the rate of growth inhibition more than 70% (Figure 6). Only one strain, *Rhodococcus* sp. IB2015I 16-1HS, was not active under the tested conditions. This could be explained by the strain ecology and requirements of this strain to specific media, including oil, paraffin waxes, or other aromatic hydrocarbons [64, 65]. The main revealed antimicrobial activity against the chosen test cultures was demonstrated by the strains *Micromonospora* sp. IB 2015I12-1, *Streptomyces* sp. IB 2015I8-1, and *Streptomyces* sp. IB 2015I9-1.

The differences observed in the activity of extracts from cell-free liquid culture and from cellular biomass within one strain can be explained by peculiarities of synthesis of natural compounds, their nature, and functional significance [34]. Also, the ability to synthesize secondary metabolites directly depends on composition of the nutrient medium used for cultivation.

As we mentioned before, microorganisms of antient and specific ecosystems and extremophilic communities are the new sources of novel antibiotics and natural compounds. Among the studies dealing with algae, a well-known example is actinobacteria related to *Nocardia* sp. ALAA 2000, which was isolated from the brown alga *Laurenica spectabilis*. The studies performed by El-Gendy et al. [66] demonstrated that this bacterial strain produces 4 antibiotics, including chrysophanol 8-methyl ether, asphodelin, justicidin B, and ayamycin. These antibiotics have pronounced antimicrobial activity against both Gram-positive and Gram-negative bacteria and against fungi, at a minimum inhibiting concentration of 0.1–10 *µ*g/ml [66].

## 5. Conclusion

During the performed study, we first time isolated several strains of cultivable actinobacteria from Baikal endemic alga *D. baicalensis*. We estimated their similarity with other actinobacteria and performed initial description of crude extracts in terms of antibiotic activity of bacteria cultivated in different nutrient media. Several strains of rare actinobacteria species were firstly found in the ecosystem of Lake Baikal and/or were shown to be involved in algal associations. The strains capable of inhibiting growth and development of bacteria and fungi are of great biotechnological and biopharmaceutical significance. Rare and active strains associated with the endemic alga *D. baicalensis* could be the promising sources for biomedical and biotechnological developments and discovery of new natural compounds.

## Figures and Tables

**Table 1:** Strains of actinobacteria isolated from *D. baicalensis* and similar ID sequences of microorganisms deposited in the GenBank, NCBI.

No	Isolate	NCBI ID	Similarity (%)	Close strains	Source, importance (if available)
1	*Nonomuraea* sp. IB 2015I9-2	MH393597	96	MF431313.1 *Nonomuraea* sp. YIM C01536	Genomic DNA isolated from cave, China
			96	KT004650.1 *Nonomuraea* sp. HCI01	Genomic DNA isolated from soil, China
			96	KC577161.1 *Nonomuraea maheshkhaliensis* SB4	Genomic DNA isolated from soil, Iran

2	*Micromonospora* sp. IB 2015I12-1	MH393594	100	MF769742.1 *Micromonospora* sp. MK-8-15	Genomic DNA isolated from sea sediments, China
			100	MF769739.1 *Micromonospora* sp. MK-8-10	Genomic DNA isolated from sea sediments, China
			100	KY858240.1 *Micromonospora chokoriensis* B032	Genomic DNA isolated from sediments, USA

3	*Sacharopolyspora* sp. IB 2015I10-2	MH393596	99	MH027604.1 *Saccharopolyspora gregorii* BM8-3	Genomic DNA isolated from sea mud, China
			99	KY056170.1 *Saccharopolyspora gregorii* DB34	Genomic DNA isolated from drug plants, India
			99	KY015031.1 *Saccharopolyspora* sp. WMMA1665	Genomic DNA isolated from sea sponges, China

4	*Rhodococcus* sp. IB 2017D16-1 HS	MH393595	98	KR817789.1 *Rhodococcus* sp. JSM 147646	Genomic DNA isolated from forest soil, China
			98	DQ157923.1 *Rhodococcus* sp. R2	Genomic DNA, USA
			98	EU878299.1 *Rhodococcus* sp. 107	Genomic DNA isolated from sediments, China

5	*Streptomyces* sp. IB 2015I8-1	MH393593	99	MH197381.1 *Streptomyces atratus* HRT13	Genomic DNA isolated from rhizosphere of *Iris pseudacorus*, Uzbekistan
			99	MF077025.1 *Streptomyces atratus* 111-LNR5	Genomic DNA isolated from deep rhizosphere desert plants, Korea
			98	MG923823.1 *Streptomyces* sp. TPML13106	Genomic DNA isolated from xylos, Korea Importance—fungal activity of extracts

6	*Streptomyces* sp. IB 2015I9-1	MH393592	95	KX928223.1 *Streptomyces* sp. AC151_JC342	Strain isolated from skin of the bat *Corynorhinus townsendii*, USA Importance—fungal activity of extracts
			95	KY000530.1 *Streptomyces* sp. Kz-12	Genomic DNA isolated from soil, India
			95	EU828543.1 *Streptomyces scabiei* 208	Strain isolated from rhizosphere of plant, China

7	*Streptomyces* sp. IB 2017D11-1	MH393590	99	KT443823.1 *Streptomyces* sp. N5-7	Strain isolated from potato, China
			99	KY243996.1 *Streptomyces glauciniger* UMBR 0137	Strain isolated from rhizosphere of mangrove forest soil, China Importance—fungal activity of extracts
			99	GQ357946.1 *Streptomyces* sp. HaNXJ11	Strain isolated from soil, China

8	*Streptomyces* sp. IB 2017D11-2	MH393591	96	EU910871.1 *Streptomyces* sp. XJ 11062	Strain isolated from soil, China. Importance—halophilic strain
			95	LN615186.1*Streptomyces* sp. MC12s29	Strain isolated from Roman's burial (etruscan burial), Spain
			95	KU323817.1 *Streptomyces* sp. YC504	Genomic DNA isolated from water, Turkey

9	*Streptomyces* sp. IB 2015I12-2	MH393589	99	MG711818.1 *Streptomyces* sp. KIB 015	Strain isolated from plant Panax Notoginseng, China. Importance—producer of Labdanmycin A and B
			99	KX928338.1 *Streptomyces* sp. AC284_FS475	Importance—fungal activity of extracts
			99	KU324437.1 *Streptomyces* sp. RJA4055	Strain isolated from excrement of earthworms that live in sewage treatment plants, China

## Data Availability

The sequence data used to support the findings of this study have been deposited in the NCBI Genbank repository (https://www.ncbi.nlm.nih.gov/genbank/). The Russian text of reports is presented in the state information system for the registration of research, experiments, and technological studies for civilian purposes (https://rosrid.ru).

## Supplementary Materials

Supplementary Materials Figure S1: microphotographs of several algae species inhabiting Lake Baikal: 1, *Mallomonas* sp.; 2, *Hannaea arcus*; 3, *Gymnodinium coeruleum*; 4, *Ceratium hirundinella*; 5, *Synedra acus*; 6, *Cyclotella minuta*. Figure S2: endemic alga *D. baicalensis.* Figure S3: phylogenetic tree of the genus *Streptomyces* sp. constructed using the maximum likelihood method based on comparison of the nucleotide sequences of 16S rRNA gene deposited in Genbank. Figure S4: phylogenetic tree of the genus *Rhodococcus* sp. constructed using the maximum likelihood method based on comparison of the nucleotide sequences of 16S rRNA gene deposited in Genbank. Figure S5: phylogenetic tree of the genus *Micromonospora* sp. constructed using the maximum likelihood method based on comparison of the nucleotide sequences of 16S rRNA gene deposited in Genbank. Figure S6: Phylogenetic tree of the genus *Saccharopolyspora* sp. constructed using the maximum likelihood method based on comparison of the nucleotide sequences of 16S rRNA gene deposited in Genbank. Figure S7: antibacterial activity of crude extracts of the strain *Streptomyces* sp. IB 2015I9-1 obtained after cultivation in different nutrient media against *St. carnosus.* Figure S8: antibacterial activity of crude extracts of the strain *Nonomuraea* sp. IB 2015I9-2 obtained after cultivation in different nutrient media against *St. carnosus.* Figure S9: antibacterial activity of crude extracts obtained from the cell-free liquid culture of the isolated strains at a concentration 25 *μ*g/L against *B. subtilis.* Figure S10: antibacterial activity of crude extracts obtained from the cellular biomass of the isolated strains at a concentration 25 *μ*g/L against *B. subtilis*. Figure S11: antibacterial activity of crude extracts obtained from the cell-free liquid culture of the isolated strains at a concentration 25 *μ*g/L against *E. coli*. Figure S12: antibacterial activity of crude extracts obtained from the cellular biomass of the isolated strains at a concentration 25 *μ*g/L against *E. coli*. Figure S13: antibacterial activity of crude extracts of *Streptomyces* sp. IB 2015I9-1 obtained after cultivation in different nutrient media against *E. coli*. Figure S14: antibacterial activity of crude extracts of *Micromonospora* sp. IB 2015I12-1 obtained after cultivation in different nutrient media against *E. coli*. Figure S15: antifungal activity of crude extracts obtained from the cell-free liquid culture of the isolated strains at a concentration 25 *μ*g/L against *S. cerevisiae*. Figure S16: antifungal activity of crude extracts obtained from the cellular biomass of the isolated strains at a concentration 25 ug/L against *S. cerevisiae*. Figure S17: antifungal activity of crude extracts of *Streptomyces* sp. IB 2015I9-1 obtained after cultivation in different nutrient media against *S. cerevisiae*. Figure S18: antifungal activity of crude extracts of *Nonomuraea* sp. IB 2015I9-2 obtained after cultivation in different nutrient media against *S. cerevisiae.*
