# Supplementary material for: Cultivable Actinobacteria First Found in Baikal Endemic Algae Is a New Source of Natural Products with Antibiotic Activity
Source: Int J Microbiol. 2020 Jul 27;2020:5359816. doi: 10.1155/2020/5359816 (PMC7403937; doi:10.1155/2020/5359816)
Supplement: Supplementary Materials — Figure S1: microphotographs of several algae species inhabiting Lake Baikal: 1, Mallomonas sp.; 2, Hannaea arcus; 3, Gymnodinium coeruleum; 4, Ceratium hirundinella; 5, Synedra acus; 6, Cyclotella minuta. Figure S2: endemic alga D. baicalensis. Figure S3: phylogenetic tree of the genus Streptomyces sp. constructed using the maximum likelihood method based on comparison of the nucleotide sequences of 16S rRNA gene deposited in Genbank. Figure S4: phylogenetic tree of the genus Rhodococcus sp. constructed using the maximum likelihood method based on comparison of the nucleotide sequences of 16S rRNA gene deposited in Genbank. Figure S5: phylogenetic tree of the genus Micromonospora sp. constructed using the maximum likelihood method based on comparison of the nucleotide sequences of 16S rRNA gene deposited in Genbank. Figure S6: Phylogenetic tree of the genus Saccharopolyspora sp. constructed using the maximum likelihood method based on comparison of the nucleotide sequences of 16S rRNA gene deposited in Genbank. Figure S7: antibacterial activity of crude extracts of the strain Streptomyces sp. IB 2015I9-1 obtained after cultivation in different nutrient media against St. carnosus. Figure S8: antibacterial activity of crude extracts of the strain Nonomuraea sp. IB 2015I9-2 obtained after cultivation in different nutrient media against St. carnosus. Figure S9: antibacterial activity of crude extracts obtained from the cell-free liquid culture of the isolated strains at a concentration 25 μg/L against B. subtilis. Figure S10: antibacterial activity of crude extracts obtained from the cellular biomass of the isolated strains at a concentration 25 μg/L against B. subtilis. Figure S11: antibacterial activity of crude extracts obtained from the cell-free liquid culture of the isolated strains at a concentration 25 μg/L against E. coli. Figure S12: antibacterial activity of crude extracts obtained from the cellular biomass of the isolated strains at a concentration 25 μg/L a [file 5359816.f1.docx]

**SUPPLEMENTARY MATERIALS**

**CULTIVABLE ACTINOBACTERIA FIRST FOUND IN BAIKAL ENDEMIC ALGAE IS A NEW SOURCE OF NATURAL PRODUCTS WITH ANTIBIOTIC ACTIVITY**

Denis V. Axenov-Gribanov^1*^, Daria V. Kostka^1,3^, Ulyana А. Vasilieva^1,4^, Zhanna M. Shatilina^1^, Maria E. Krasnova^1^, Ekaterina V. Pereliaeva^1^, Elena D. Zolotovskaya^1^, Maria M. Morgunova^1^, Olga O. Rusanovskaya^1^, Maxim A. Timofeyev^1,2^

^1^ Irkutsk State University, Karl Marx St. 1, 664003, Irkutsk, Russia

^2^ Baikal Research Centre, Lenin str., 21, 664003, Irkutsk, Russia

^3^ Irkutsk Regional Clinical Advisory and Diagnostic Center, Baykalskaya str., 109, Irkutsk, Russia

^4^ Siberian Institute of Plant Physiology and Biochemistry, 132 Lermontov str., 664033, Irkutsk, Russia

* Author for correspondence

Dr. Denis Axenov-Gribanov

664025, Irkutsk

3 Lenin str.

Tel.: +7 (950) 065-84-55

Tel//Fax: +7 (3952) 24-30-77 (+116)

*e-mail: denis.axengri@gmail.com*

**Abstract**

Inadequate use of antibiotics has led to spread of microorganisms resistant to effective antimicrobial compounds for humans and animals. This study was aimed to isolate cultivable strains of actinobacteria associated with Baikal endemic alga *Draparnaldioides baicalensis* and estimate their antibiotic properties.

During this study, we isolated both widespread and dominant strains related to the genus *Streptomyces* and representatives of the genera *Saccharopolispora, Nonomuraea, Rhodococcus* and *Micromonospora*. For the first time, actinobacteria belonging to the genera *Nonomuraea* and *Saccharopolispora* were isolated from Baikal ecosystem. Also, it was the first time when actinobacteria of the genus *Nonomuraea* were isolated from freshwater algae. Some rare strains demonstrated activity inhibiting growth of bacteria and yeasts. Also, it has been shown that the strains associated with Baikal alga *D. baicalensis* are active against both Gram-positive and Gram-negative bacteria. According to this study and previously published materials, diversity of cultivable actinobacteria and rare strains isolated from *D. baicalensis* is comparable to that of cultivable actinobacteria previously isolated from plant sources of Lake Baikal. Also, it exceeds the cultivable actinobacteria diversity previously described for macroinvertebrates, water or sediments of Lake Baikal. The large number of rare and active strains associated with the endemic alga *D. baicalensis* could be the promising sources for biopharmaceutical and biotechnological developments and discovery of new natural compounds.

**Key words:** Actinobacteria, algae, antimicrobial natural products, Baikal, *Draparnaldioides* sp.


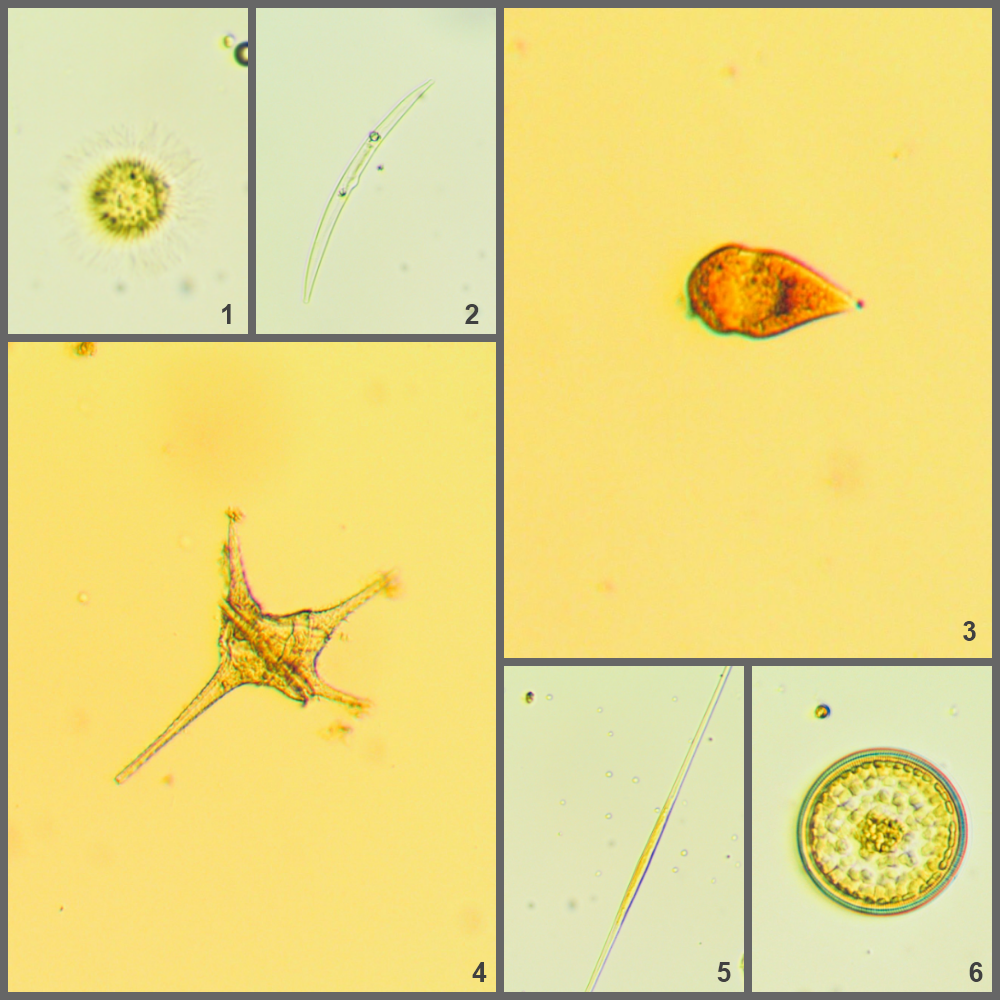


**Figure S1.** Microphotographs of several algae species inhabiting Lake Baikal: 1 - *Mallomonas* sp.; 2 - *Hannaea arcus*; 3 - *Gymnodinium coeruleum*; 4 - *Ceratium hirundinella*; 5 - *Synedra acus*; 6- *Cyclotella minuta*.

**
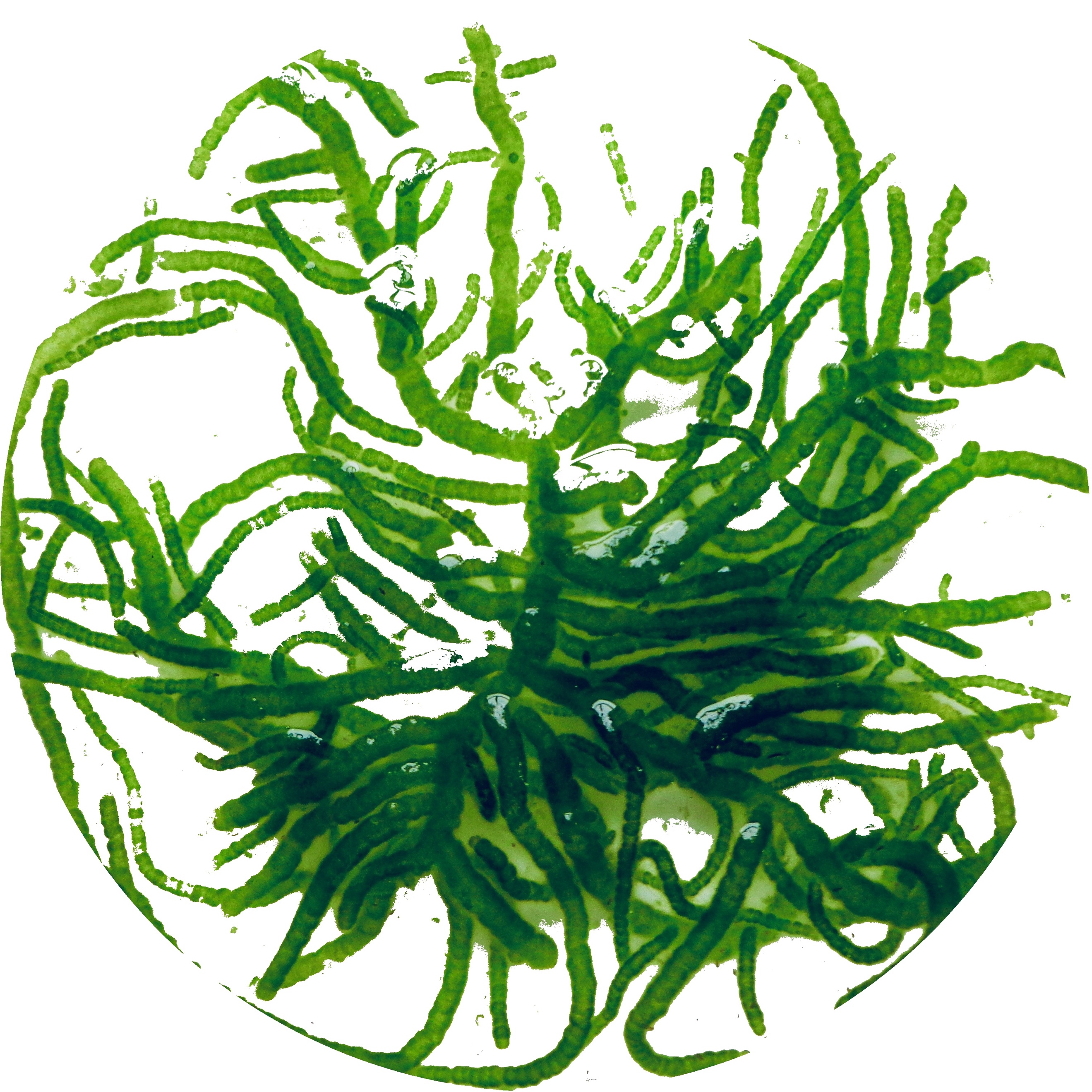
**

**Figure S2.** Endemic alga *D. baicalensis.*

**Figure S3.** Phylogenetic tree of the genus *Streptomyces* sp. constructed using the Maximum likelihood method based on comparison of the nucleotide sequences of 16S rRNA gene deposited in Genbank. *Note -Bootstrap – 1000. The analysis involves 47 sequences, length of nucleotides - 411. The tree includes 11 strains of bacteria isolated from soil (marked “from soil”); 13 strains of bacteria isolated from water of different reservoirs (marked “from water”); 13 strains of bacteria isolated from algae (marked “from algae”); 5 strains of bacteria isolated from Lake Baikal (marked “from Lake Baikal”) and 5 strains isolated in the course of this study. The analysis was carried out in MEGA7 program. Outgroup is presented by B. licheniformis.*

**Figure S4.** Phylogenetic tree of the genus *Rhodococcus* sp. constructed using the Maximum likelihood method based on comparison of the nucleotide sequences of 16S rRNA gene deposited in Genbank. *Note - Bootstrap – 1000. The analysis involves 27 sequences, length of nucleotides - 411. The tree includes 9 strains of bacteria isolated from soil (marked “from soil”); 9 strains of bacteria isolated from water of different reservoirs (marked “from water”); 3 strains of bacteria isolated from algae (marked “from algae”); 5 strains of bacteria isolated from Lake Baikal (marked “from Lake Baikal”) and 1 strain isolated in the course of this study. The analysis was carried out in MEGA7 program. Outgroup is presented by B. licheniformis.*

**Figure S5.** Phylogenetic tree of the genus *Micromonospora* sp. constructed using the Maximum likelihood method based on comparison of the nucleotide sequences of 16S rRNA gene deposited in Genbank. *Note - Bootstrap – 1000. The analysis involves 21 sequences, length of nucleotides - 411. The tree includes 7 strains of bacteria isolated from soil (marked “from soil”); 7 strains of bacteria isolated from water of different reservoirs (marked “from water”); 1 strain of bacteria isolated from algae (marked “from algae”); 5 strains of bacteria isolated from Lake Baikal (marked “from Lake Baikal”) and 1 strain isolated in the course of this study. The analysis was carried out in MEGA7 program. Outgroup is presented by B. licheniformis.*

**Figure S6.** Phylogenetic tree of the genus *Saccharopolyspora* sp. constructed using the Maximum likelihood method based on comparison of the nucleotide sequences of 16S rRNA gene deposited in Genbank. *Note - Bootstrap – 1000. The analysis involves 24 sequences, length of nucleotides - 411. The tree includes 11 strains of bacteria isolated from soil (marked “from soil”); 11 strains of bacteria isolated from water of different reservoirs (marked “from water”); 1 strain of bacteria isolated from algae (marked “from algae”); and 1 strain isolated in the course of this study. The analysis was carried out in MEGA7 program. Outgroup is presented by B. licheniformis.*

**Figure S7.** Antibacterial activity of crude extracts of the strain *Streptomyces* sp. IB 2015I9-1 obtained after cultivation in different nutrient media against *St. carnosus.* *Note “-” - Inhibition rate, in %, Mean ± SD.*

**Figure S8.** Antibacterial activity of crude extracts of the strain *Nonomuraea* sp. IB 2015I9-2 obtained after cultivation in different nutrient media against *St. carnosus. Note “-” - Inhibition rate, in %, Mean ± SD.*

**Figure S9.** Antibacterial activity of crude extracts obtained from the cell-free liquid culture of the isolated strains at concentration 25 ug/L against *B. subtilis. Note “-” - Inhibition rate, in %, Mean ± SD.*

**Figure S10.** Antibacterial activity of crude extracts obtained from the cellular biomass of the isolated strains at concentration 25 ug/L against *B. subtilis. Note “-” - Inhibition rate, in %, Mean ± SD.*

**Figure S11.** Antibacterial activity of crude extracts obtained from the cell-free liquid culture of the isolated strains at concentration 25 ug/L against *E. coli. Note “-” - Inhibition rate, in %, Mean ± SD.*

**Figure S12.** Antibacterial activity of crude extracts obtained from the cellular biomass of the isolated strains at concentration 25 ug/L against *E. coli. Note “-” - Inhibition rate, in %, Mean ± SD.*

**Figure S13.** Antibacterial activity of crude extracts of *Streptomyces* sp. IB 2015I9-1 obtained after cultivation in different nutrient media against *E. coli. Note “-” - Inhibition rate, in %, Mean ± SD.*

**Figure S14.** Antibacterial activity of crude extracts of *Micromonospora* sp. IB 2015I12-1 obtained after cultivation in different nutrient media against *E. coli. Note “-” - Inhibition rate, in %, Mean ± SD.*

**Figure S15.** Antifungal activity of crude extracts obtained from the cell-free liquid culture of the isolated strains at concentration 25 ug/L against *S. cerevisiae. Note “-” - Inhibition rate, in %, Mean ± SD.*

**Figure S16.** Antifungal activity of crude extracts obtained from the cellular biomass of the isolated strains at concentration 25 ug/L against *S. cerevisiae. Note “-” - Inhibition rate, in %, Mean ± SD.*

**Figure S17.** Antifungal activity of crude extracts of *Streptomyces* sp. IB 2015I9-1obtained after cultivation in different nutrient media against *S. cerevisiae. Note “-” - Inhibition rate, in %, Mean ± SD.*

**Figure S18.** Antifungal activity of crude extracts of *Nonomuraea* sp. IB 2015I9-2 obtained after cultivation in different nutrient media against *S. cerevisiae. Note “-” - Inhibition rate, in %, Mean ± SD.*
